# Supplementary material for: TFIIS is required for reproductive development and thermal adaptation in barley
Source: Plant Cell Rep. 2024 Oct 10;43(11):260. doi: 10.1007/s00299-024-03345-1 (PMC11467006; doi:10.1007/s00299-024-03345-1)
Supplement: Supplementary file 3 — Supplementary file3 (PDF 8020 KB) [file 299_2024_3345_MOESM3_ESM.pdf]

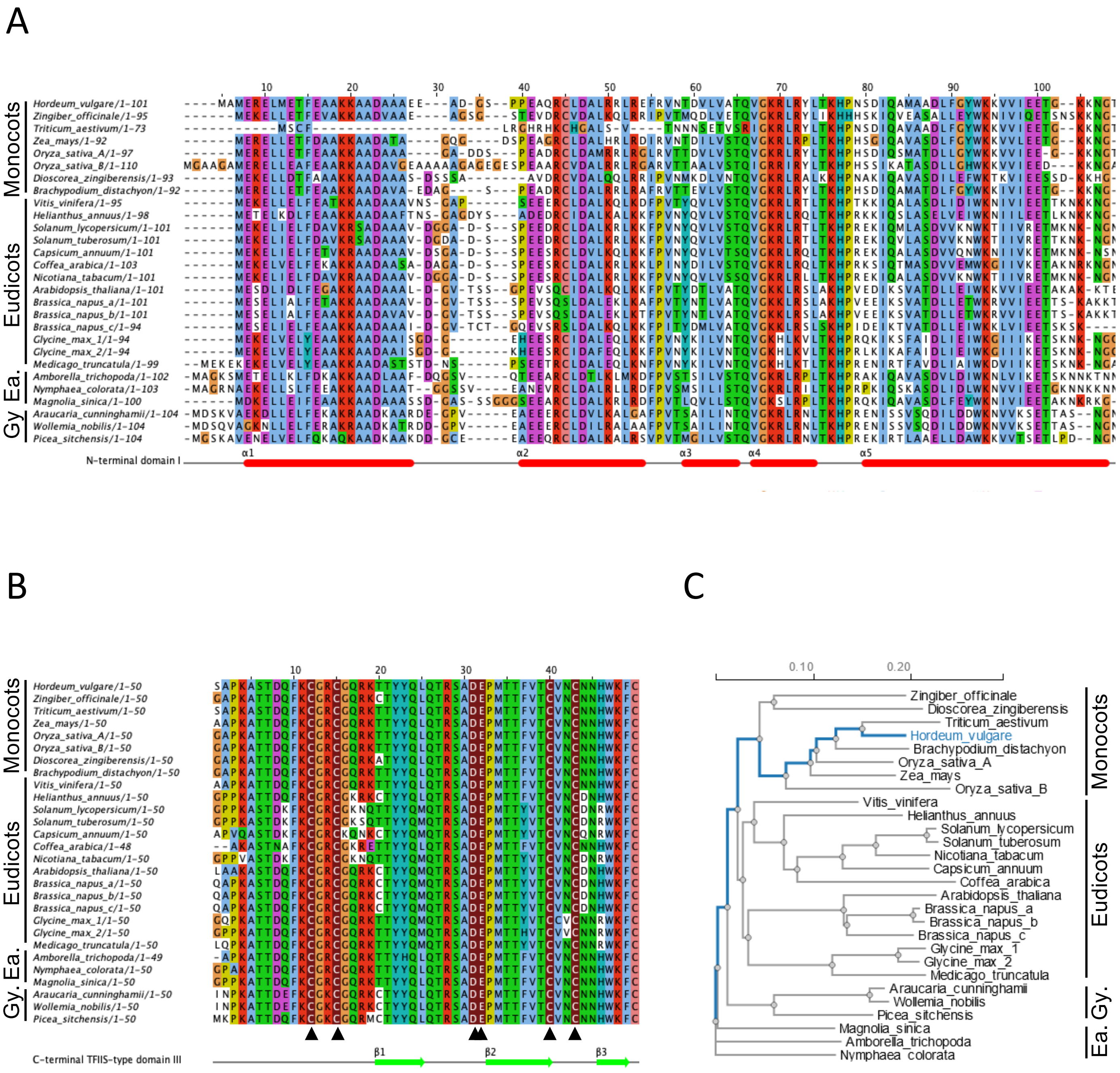

Supplementary figure S1: Protein alignment of TFIIS protein homologs; The sequences of gymnosperm (Gy), early dicot (Ea), eudicot and monocot TFIIS protein homologs (as shown on left) were aligned by Jalview ClustalO tool and secondary structure α-helices and $\boldsymbol{\beta}$-sheets predicted (shown on bottom) for TFIIS domain I (A) or TFIIS domain III (B). The four cystein residues forming the zinc-finger and acidfic dipeptide of TFIIS domain III is shown with arrowheads below. (C) Phylogenetic tree of TFIIS homolgs in representative species of plant kingdom; the length of the branches is indicative for evolutionary distance between protein sequences.

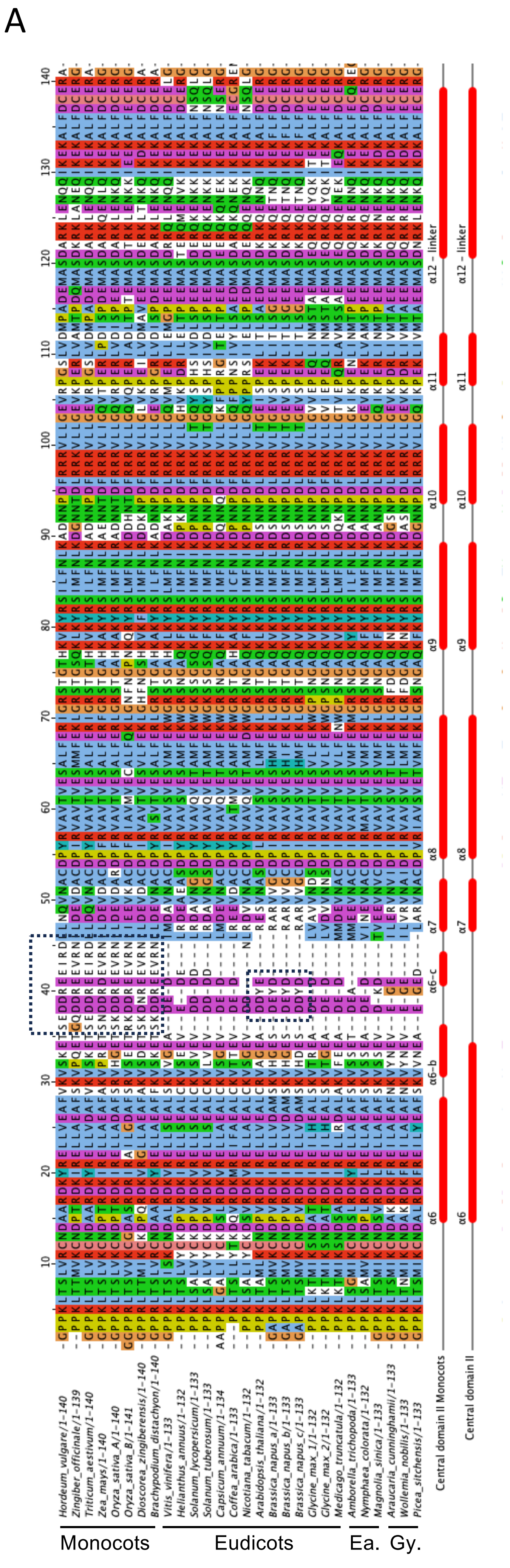

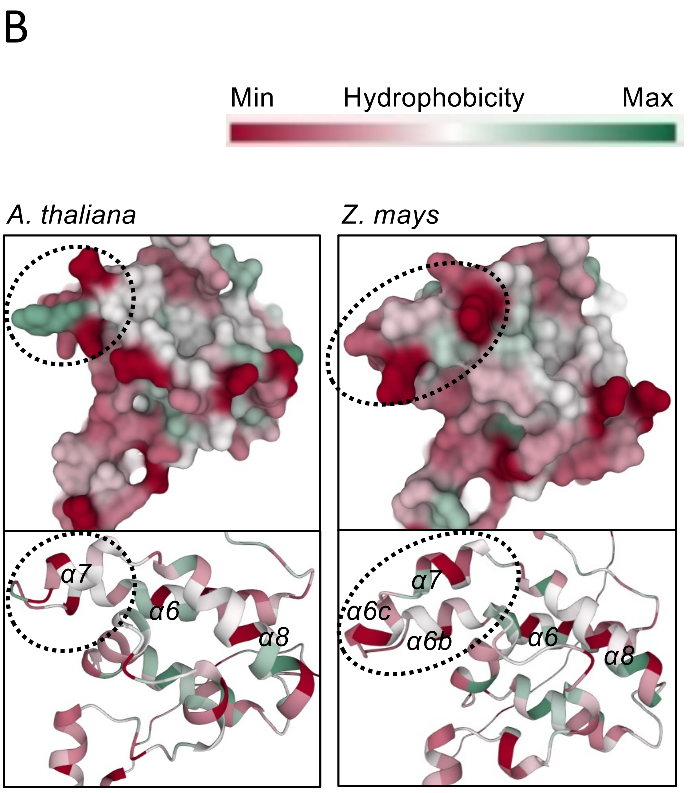

Supplementary figure S2: Protein alignment of domain II of TFIIS protein homologs. (A) The sequences of gymnosperm (Gy), early dicot (Ea), eudicot and monocot TFIIS protein homologs (as shown on bottom) were aligned by Jalview ClustalO tool and secondary structure α-helices predicted (shown on the right) for TFIIS domain II. (B) The *in silico* 3D structure prediction of TFIIS domain II α6-to-α8 region of *A. thaliana* and *Zea mays* homologs; hydrophobicity heatmap color legend shown on top; the Arabidopsis-specific and Z. mays (monocot)-specific structural alterations are highlighted by dotted squares (A) and dotted circles (B).

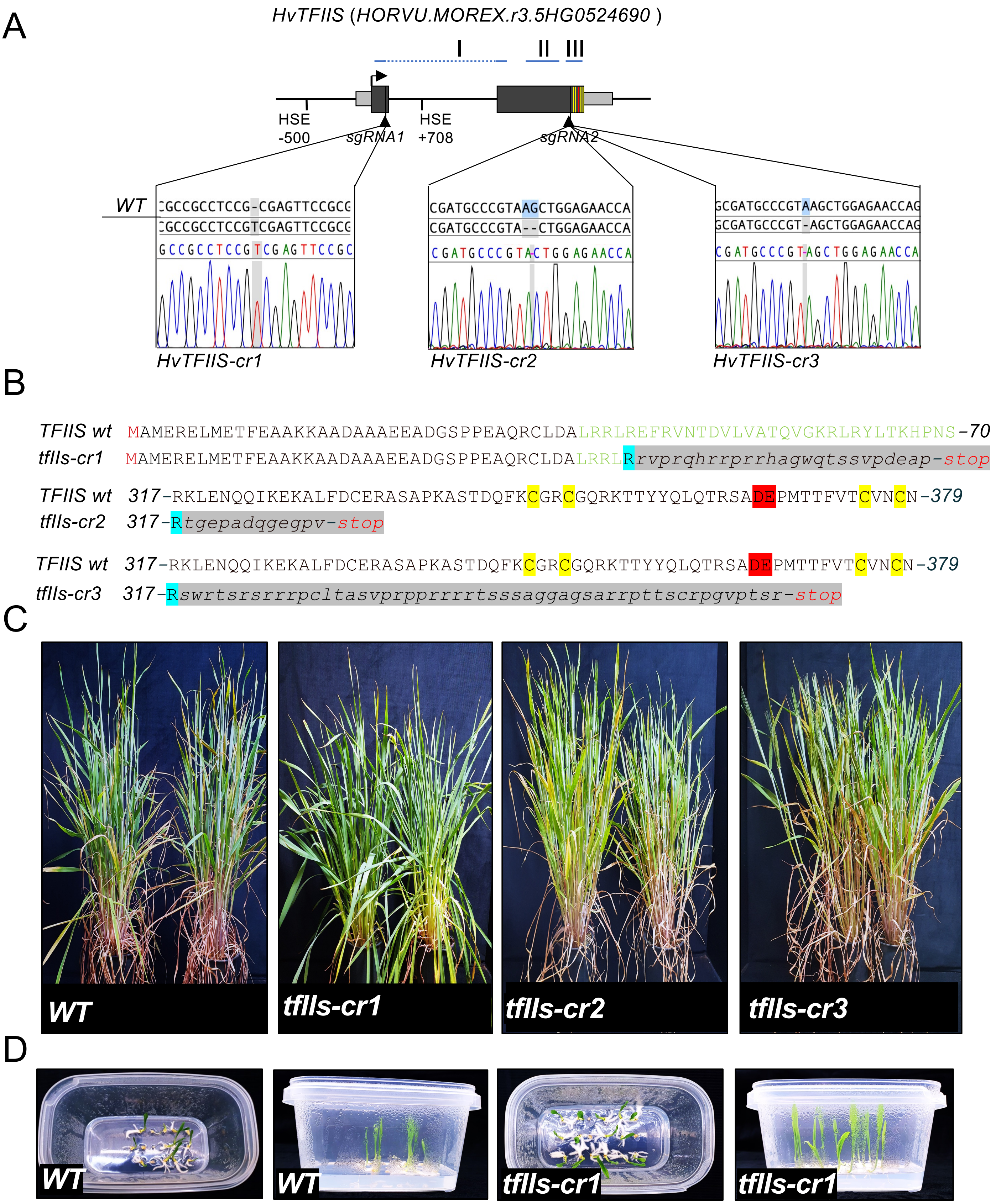

Supplementary figure S3: TFIIS is negligible for vegetative growth but needed for reproductive development in barley. (A) (A) Schematic representation of *HvTFIIS* gene locus; protein domain I, II and III location is shown above; exons as black boxes, UTRs as grey boxes, Zn finger domain cysteine residues and DE acidic dipeptide motif as yellow and red line, respectively; HSE *cis* elements and location of sgRNA guide sites are shown below; (B) TFIIS mutants of barley were created by CRISPR mutagenesis; Insertion or deletion mutation within the TFIIS locus in the selected transgenic lines are shown on chromatograms; (C) HvTFIIS protein amino-acid changes with premature termination codon is shown below; last correct aminoacid is highlighted with cyan, changed amino acid sequence with grey, Zn-finger motif residues with yellow, catalytic DE dipeptide with red; Vegetative growth of wild tpye (wt) and CRISPR mutant *tfIIs-cr1*, *-cr2* and *-cr3* barley plants; *tfIIs-cr1* plants are slightly shorter in stature, while *tfIIs-cr2* and *-cr3* plants are indistinguishable to wt plants; (D) Embryo rescue of *tfIIs-cr1* seeds, wild-type and mutant embryos were extracted from the seeds and placed on sterile germinating agar medium for growth; wt and mutant embryos were treated similarly for generation of plants and TMHT treatment work shown in Fig 2 and S4.

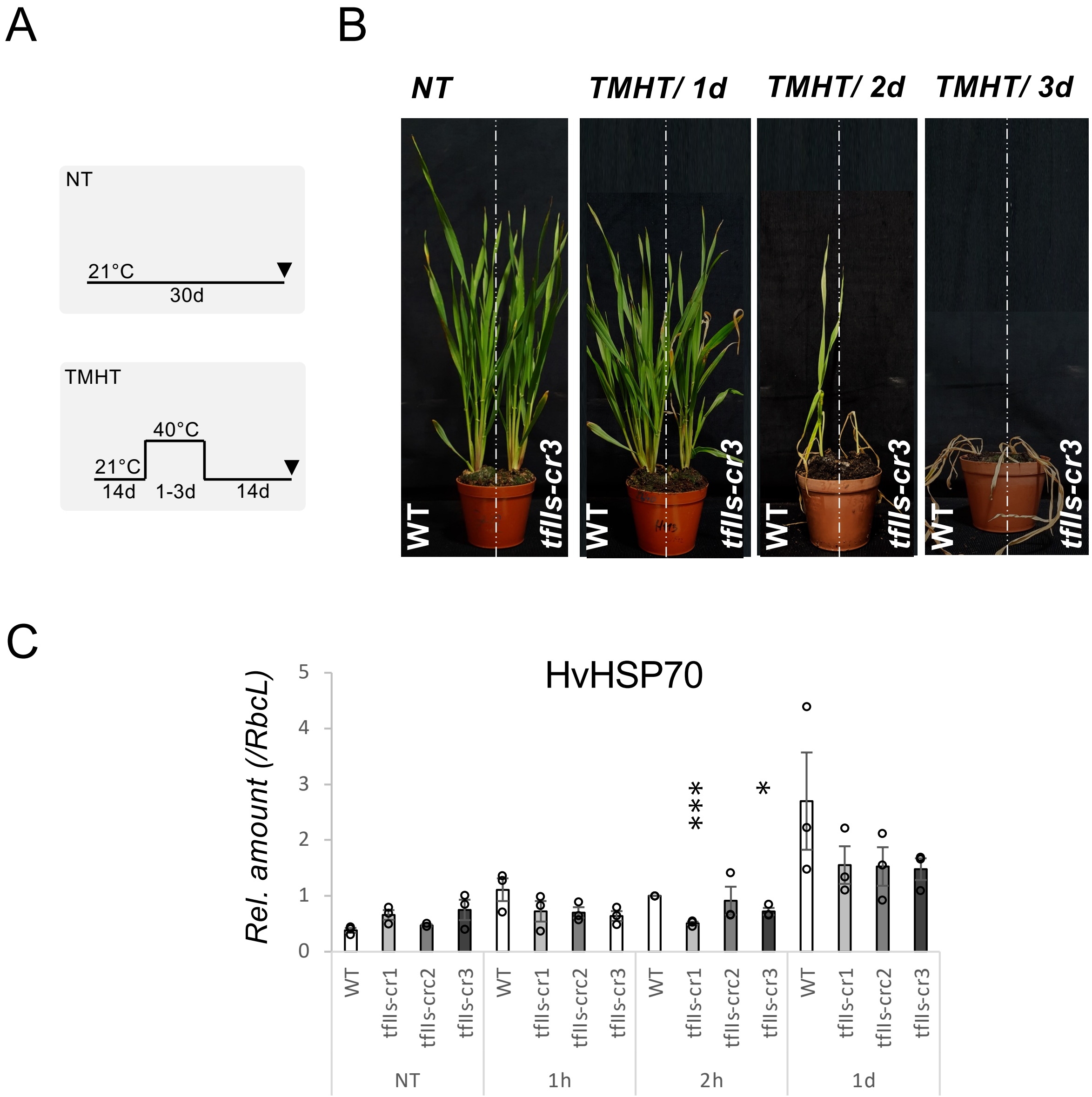

Supplementary figure S4: TFIIS is indispensable for efficient heat stress response. (A) Schematic representation of non-treated (NT) and Thermotolerance to Moderately High Temperature regime (TMHT); arrowheads show the time of sampling; (B) Wild-type (wt) and *tfIIs* CRISPR mutant *tfIIs-cr3* were exposed to NT or TMHT for 1 hour (1h), 2h or 1 day (1d); (C) Quantification of HSP70 protein amount changes in response to heat stress during a timeseries; bars represent standard errors based on three bio reps; P-values based on two-tailed Student's t-test (*P < 0.05, **P < 0.01, ***P < 0.001) show differences between wild type and mutant plants.

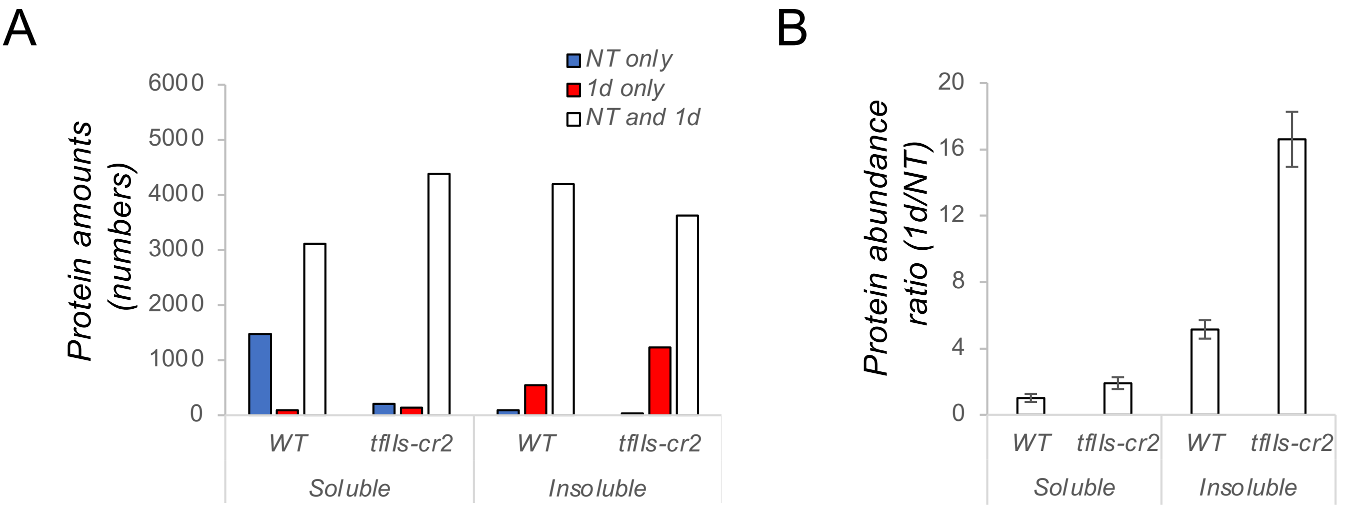

C

	Soluble				Insoluble			
	WT		tfIIs-cr2		WT		tfIIs-cr2	
source	Terms	P values (adj.)	Terms	P values (adj.)	Terms	P values (adj.)	Terms	P values (adj.)
GO:MF			hydrolase activity	1,3E-02	catalytic activity	1,1E-02	catalytic activity	7,7E-06
GO:MF					isomerase activity	3,2E-02	hydrolase activity	3,8E-03
GO:MF							hydro-lyase activity	1,9E-02
GO:MF							peptide deformylase activity	5,0E-02
GO:MF							RNA 7-methylguanosine cap binding	5,0E-02
GO:BP					small molecule metabolic process	1,5E-04	small molecule metabolic process	5,1E-07
GO:BP							macromolecule localization	4,0E-03
GO:BP							positive regulation of mRNA splicing, via spliceosome	5,9E-03
GO:BP							positive regulation of RNA splicing	5,9E-03
GO:BP							positive regulation of mRNA processing	5,9E-03
GO:BP							organonitrogen compound biosynthetic process	7,8E-03
GO:BP							protein transport	9,3E-03
GO:BP							nucleobase-containing small molecule metabolic process	1,0E-02
GO:BP							cellular localization	1,6E-02
GO:BP							vesicle-mediated transport	1,7E-02
GO:BP							cellular macromolecule localization	1,8E-02
GO:BP							protein localization	1,8E-02
GO:BP							positive regulation of mRNA metabolic process	2,0E-02
GO:BP							organic substance transport	2,1E-02
GO:BP							organophosphate metabolic process	2,3E-02
GO:BP							aspartate family amino acid metabolic process	2,4E-02
GO:BP							establishment of protein localization	3,5E-02
GO:BP							establishment of localization in cell	3,6E-02
GO:BP							organophosphate biosynthetic process	4,3E-02
GO:CC			chloroplast	4,9E-03			cytoplasm	1,1E-05
GO:CC			plastid	9,2E-03			plastid	1,3E-02
GO:CC			cytoplasm	1,7E-02			organelle envelope	1,4E-02
GO:CC							envelope	1,4E-02
GO:CC							organelle inner membrane	1,7E-02
GO:CC							eukaryotic translation initiation factor 4F complex	5,0E-02
GO:CC							cytoplasmic stress granule	5,0E-02

Supplementary figure S5: Insoluble protein aggregate accumulation in response to heat stress in wt and *tfIIs-cr2* plants. (A) Mass spectroscopy analysis of soluble and insoluble protein fractions of wt and *tfIIs-cr2* mutant barley plants, samples as shown in the legend top-right; (B) Average abundance ratio of proteins found in both non-treated (NT) and heat-treated (1d) samples; error bars are calculated based on protein ratio averages; (C) Gene ontology (*GO*) terms of MS detected proteins found heat-treated samples of wt and *tfIIs-cr2* mutant plants.

Supplementary Table 1: DNA oligonucleotide primers used in the study.

Crispr constructs	
HvTFIIS_cr1_F	GGC GGT TGA CGC GGA ACT CGC GG
HvTFIIS_cr1_R	AAA CCC GCG AGT TCC GCG TCA AC
HvTFIIS_cr2_F	GGC GGC GAG CGA TGC CCG TAA GC
HvTFIIS_cr2_R	AAA CGC TTA CGG GCA TCG CTC GC
Genotyping	
HvTFIIS_crispr1_check_F	CTG CGC CTA GGG TTT TCA TC
HvTFIIS_crispr1_check_R	GCT TTC CGC ACC TCT CAG TC
HvTFIIS_crispr2_check_F	ACA GTG GAG TCG GCC TTG TT
HvTFIIS_crispr2_check_R	TAC GAA AAA CCG AGC CAA CC
HvTFIIS_cr1_check-gF	GGA GAG GGA GCT GAT GGA GA
HvTFIIS_cr1_check-gR	CGA TAG GGC ACG GAT AGT CTG
qrtPCR	
HvTFIIS_qF:	TCG CCA CGC AGG TTG GCA AAC G
HvTFIIS_qR:	TTC AAT AAC AAC CTT CTT CCA G
HvActin7_qF	CGT GTT GGA TTC TGG TGA TG
HvActin7_qR	AGC CAC ATA TGC GAG CTT CT
HvHSPc70-4_qF	CAA CAC CGT TTT TGA TGC CA
HvHSPc70-4_qR	ACC ACG ATC ATC GGC TTG T
HvHSP90_qF	AAC TCA TCT GAC GCG CTT GA
HvHSP90_qR	GCT GTC GAT GAT GGA GAG CGT
HvHSP101-1_qF	GTC ATG CAG GAG GTG AGG AGG
HvHSP101-1_qR	CCA CGT CCT TCA TCT GCA G
